# Glycopeptide Antibiotic Resistance Genes: Distribution and Function in the Producer Actinomycetes

**DOI:** 10.3389/fmicb.2020.01173

**Published:** 2020-06-17

**Authors:** Oleksandr Yushchuk, Elisa Binda, Flavia Marinelli

**Affiliations:** Department of Biotechnology and Life Sciences, University of Insubria, Varese, Italy

**Keywords:** antimicrobial resistance, glycopeptide antibiotics, *van* genes, glycopeptide producers, biosynthetic gene clusters

## Abstract

Glycopeptide antibiotics (GPAs) are considered drugs of “last resort” for the treatment of life-threatening infections caused by relevant Gram-positive pathogens (enterococci, staphylococci, and clostridia). Driven by the issue of the never-stopping evolution of bacterial antibiotic resistance, research on GPA biosynthesis and resistance is developing fast in modern “post-genomic” era. It is today widely accepted that resistance mechanisms emerging in pathogens have been acquired from the soil-dwelling antibiotic-producing actinomycetes, which use them to avoid suicide during production, rather than being orchestrated *de novo* by pathogen bacteria upon continued treatment. Actually, more and more genomes of GPA producers are being unraveled, carrying a broad collection of differently arranged GPA resistance (named *van*) genes. In the producer actinomycetes, *van* genes are generally associated with the antibiotic biosynthetic gene clusters (BGCs) deputed to GPA biosynthesis, being probably transferred/arranged together, favoring a possible co-regulation between antibiotic production and self-resistance. GPA BGC-associated *van* genes have been also found mining public databases of bacterial genomic and metagenomic sequences. Interestingly, some BGCs for antibiotics, seemingly unrelated to GPAs (e.g., feglymycin), carry *van* gene homologues. Herein, we would like to cover the recent advances on the distribution of GPA resistance genes in genomic and metagenomics datasets related to GPA potential/proved producer microorganisms. A thorough understanding of GPA resistance in the producing microorganisms may prove useful in the future surveillance of emerging mechanisms of resistance to this clinically relevant antibiotic class.

## Gpa Mode of Action and Resistance Genes in Gram-Positive Pathogens

According to a recent report (WHO, 2017), drug-resistant infections will kill more people than cancer in just over three decades: by 2050, 10 million people are going to die every year due to antimicrobial resistance (AMR). Consequently, it is mandatory to stimulate discovery and development of novel antibiotics to counteract AMR (O’Neill, 2016). Glycopeptide antibiotics (GPAs) are frequently used to treat life-threatening infections caused by multidrug-resistant Gram-positive pathogens, such as *Staphylococcus aureus*, *Enterococcus* spp., and *Clostridium difficile* (for a review on their discovery and development, see Marcone et al., 2018; on their antimicrobial activity and clinical use, Zeng et al., 2016). GPAs inhibit bacterial cell wall synthesis in Gram-positive bacteria by binding to d-alanyl-d-alanine (d-Ala-d-Ala) dipeptide terminus of peptidoglycan (PG) precursors, sequestering the substrate from transpeptidation and transglycosylation reactions in the late extracellular stages of PG cross-linking. Thus, GPA action ultimately results in destabilizing cell wall integrity, causing bacterial cell death (Perkins and Nieto, 1974). Gram-negative microorganisms are intrinsically resistant to GPAs, because of their outer membrane, which prevents these molecules entering into the periplasm. In Gram-positive bacteria, the onset of vancomycin resistance was long-delayed in comparison to other antibiotic classes. The first vancomycin-resistant clinical isolate – an *Enterococcus faecium* strain – was reported in 1987, more than 30 years after the clinical introduction of vancomycin (Leclercq et al., 1988, Miller et al., 2016). Unfortunately, today a vast majority of *E. faecium* isolates harbor vancomycin resistance genes (*van*) (Vehreschild et al., 2019). The first vancomycin-resistant *S. aureus* (VRSA) isolate was reported in 2002 as a result of horizontal gene transfer from resistant enterococci (Bartley, 2002; Weigel et al., 2003); nowadays, 52 VRSA strains have been described worldwide (Cong et al., 2020).

The GPA resistance mechanisms in Gram-positive pathogens were intensively studied starting from the pioneering work published in the 1990s (Arthur et al., 1992, 1996). Gram-positive pathogens escape GPA action by reprogramming PG precursor biosynthesis, replacing the terminal d-Ala with d-lactate (d-Ala-d-Lac) or d-serine (d-Ala-d-Ser), thus reducing the affinity for cellular targets (Arthur et al., 1992, 1996; Courvalin, 2006). In enterococci, many different GPA-resistant phenotypes have been described according to their *van* gene operon organization (for a review, see Binda et al., 2014): in *vanA*, *vanB*, *vanD*, and *vanM* the key ligase determines the replacement of the terminus d-Ala with d-Lac, whereas in *vanC*, *vanE*, *vanG*, *vanL*, and *vanN* d-Ala. The d-Ala-d-Lac-type operons are located either on plasmids or on chromosomes, whereas the d-Ala-d-Ser-type ones are exclusively on the bacterial chromosome, except the case of *vanN* found on a plasmid in *E. faecium*. Operon expression could be inducible by GPAs (*vanA*, *vanB*, *vanG*, *vanE*, *vanL*, and *vanM*) or constitutive (*vanC*, *vanD*, and *vanN*) (Reynolds and Courvalin, 2005; Depardieu et al., 2007; Binda et al., 2014). The most clinically relevant manifestation of GPA resistance occurs in VanA enterococci and staphylococci, and in VanB enterococci. The first group is highly resistant to both vancomycin and teicoplanin, whereas the second group only to vancomycin. In both of them, resistance is mediated by the GPA-induced expression of the transposone-located *vanHAX* gene operon under the transcriptional control of the VanR/VanS two-component system (TCS). VanS is a membrane-associated sensor that in VanA bacteria is activated by the presence of either vancomycin or teicoplanin, whereas in VanB it is activated only by vancomycin. Consequently, VanB enterococci are sensitive to teicoplanin (Arthur et al., 1997, 1999; Arthur and Quintiliani, 2001). Activated VanS transfers a phosphoryl group to VanR, which is the response regulator that controls the co-transcription of the *vanH*, *vanA*, *vanX*, and *vanY* genes (Wright et al., 1993; Arthur et al., 1997, 1999; Arthur and Quintilliani, 2001). VanH is a dehydrogenase that reduces pyruvate to d-lactate; VanA is the key ligase that catalyzes the formation of the d-Ala-d-Lac resistant depsipeptide (Bugg et al., 1991; Arthur et al., 1992); VanX is a d,d-dipeptidase, which removes the intracellular pool of d-Ala-d-Ala produced by the native enterococcal ligase, ensuring that d-Ala-d-Lac is incorporated into PG precursors (Reynolds et al., 1994; Wu et al., 1995); and finally VanY has an ancillary role as a d,d-carboxypeptidase cleaving the last d-Ala from the residual pentapeptide PG precursors terminating in d-Ala-d-Ala (Arthur et al., 1998). Among the d-Ala-d-Ser-type operons, the better investigated was the *vanC*. It encodes for a racemase (VanT) that converts l-Ser to d-Ser, a ligase (VanC) that synthesizes d-Ala-d-Ser, and a bi-functional d,d-dipeptidase/d,d-carboxypeptidase (VanXYc) that cleaves the residual pools of d-Ala-d-Ala (Billot-Klein et al., 1994; Reynolds and Courvalin, 2005). In VanC phenotype, the TCS VanRcSc is located downstream the operon, but the resistance is constitutive due to mutations in the sensor VanSc (Healy et al., 2000; Hong et al., 2008; Koteva et al., 2010). VanC enterococci are intrinsically resistant to low levels of vancomycin, although they remain sensitive to teicoplanin.

Additional variants of these *van* gene operons were found in other Gram-positive pathogens including *Listeria* spp., streptococci, clostridia (Biavasco et al., 1996; Poyart et al., 1997; Peltier et al., 2013), and also in nonpathogenic Gram-positives, including *Bacillus circulans*, *Oerskovia* spp., *Corynebacterium* spp., and *Streptomyces coelicolor* (Power et al., 1995; Fontana et al., 1997; Hong et al., 2004). A novel vancomycin *vanF* operon (*vanY_F_Z_F_H_F_FX_F_*) was described in *Paenibacillus popilliae*, an environmental bacteria used as biopesticide to counteract beetle larvae that caused milky disease in Japan (Patel et al., 2000; Ahmed and Baptiste, 2018). The dissemination of GPA resistance more recently reached zoonotic pathogens such as the emergent *Streptococcus suis*, where the low level of vancomycin-resistance is due to the presence of a *vanG*-like operon (Huang et al., 2018). Herein, we focus our attention on *van* genes distribution and function in the GPA-producing actinomycetes, which are considered the putative primary source of the variety of GPA-resistant determinants occurring in environmental bacteria and pathogens (Marshall et al., 1998; Beltrametti et al., 2007; Marcone et al., 2010, 2014; Schäberle et al., 2011).

## Updating The Glycopeptide Resistance Paradigm For The Gpa-Producing Strains: *Van* Genes And Their Organization In Known And Putative Gpa Bgcs

**Figure 1(Continued)FIGURE 1:**
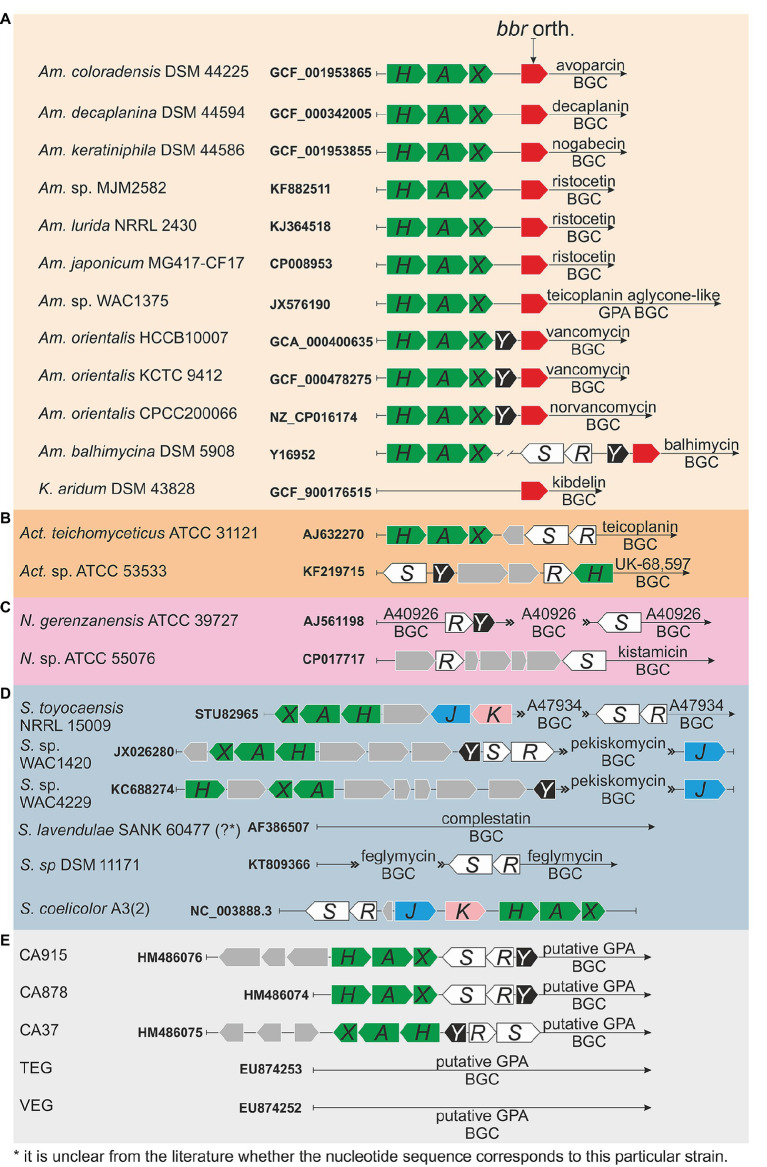
Organization of *van* genes orthologues in glycopeptide antibiotic (GPA) biosynthetic gene clusters (BGCs) of producers and metagenomics sequences: (A) family *Pseudonocardiaceae*: *Amycolatopsis coloradensis* DSM 44225 (GCF_001953865) – avoparcin producer (Kunstmann et al., 1968; Labeda, 1995); *Amycolatopsis decaplanina* DSM 44594 (GCF_000342005, Kaur et al., 2013) – decaplanin producer (Sanchez et al., 1992); *Amycolatopsis keratiniphila* subsp. *nogabecina* DSM 44586 (=*Amycolatopsis keratiniphila* subsp. *nogabecina* FH1893, GCF_001953855) – nogabecin producer (Wink et al., 2003); *Amycolatopsis* sp. MJM2582 (KF882511, Truman et al., 2014), *Amycolatopsis lurida* NRRL2430 (KJ364518, Truman et al., 2014), *Amycolatopsis japonicum* MG417-CF17 (CP008953, Spohn et al., 2014) – ristocetin producers; *Amycolatopsis* sp. WAC4169 (JX576190, Thaker et al., 2013) – producer of teicoplanin aglycone-like GPA; *Amycolatopsis orientalis* HCCB10007 (=*Am. keratiniphila*, GCA_000400635, Xu et al., 2014) and *Amycolatopsis orientalis* KCTC 9412 (=*Am. orientalis* DSM 40040, GCF_000478275, Jeong et al., 2013) – vancomycin producers; *Amycolatopsis orientalis* CPCC200066 (=*Am. orientalis* B-37, NZ_CP016174, Lei et al., 2015) – norvancomycin producer; *Amycolatopsis balhimycina* DSM 5908 (Y16952, Shawky et al., 2007) – balhimycin producer (Nadkarni et al., 1994; Wink et al., 2003); *Kibdelosporangium aridum* DSM 43828 (GCF_900176515) – the producer of kibdelins (Shearer et al., 1985), (B) genus *Actinoplanes* (please see main text for more details), (C) genus *Nonomuraea* (please see main text for more details), (D) genus *Streptomyces* (please see main text for more details); although *S. coelicolor* is not a GPA producer, organization of *S. coelicolor van* gene orthologues is also given, and (E) metagenomics sequences (please see main text for more details).

Actinomycetes are Gram-positive soil-dwelling bacteria, which produce about two-thirds of the naturally derived antibiotics with clinical use (Bérdy, 2012; Barka et al., 2015), including GPAs (Nicolaou et al., 1999). Clinically relevant GPAs are produced by *Amycolatopsis orientalis* (vancomycin), *Actinoplanes teichomyceticus* (teicoplanin), and *Nonomuraea gerenzanensis* (dalbavancin precursor – A40926) (Zeng et al., 2016; Marcone et al., 2018). GPA producers require self-resistance mechanisms to avoid suicide during antibiotic production and, like in pathogens, such resistance is due to *van* genes, whose description dates back to the end of the 1990s, one decade later than in pathogens (Marshall et al., 1997, 1998). Sequence and operon structure similarities of *van* genes between pathogens and GPA-producers are significant (Hong et al., 2008; Binda et al., 2014). The intriguing aspect is that in GPA producers, *van* genes are usually located within the GPA biosynthetic gene clusters (BGCs) deputed to the antibiotic biosynthesis (Pootoolal et al., 2002; Beltrametti et al., 2007; Marcone et al., 2010, 2014; Schäberle et al., 2011). In the last two decades, multiple novel GPA BGCs from actinomycetes were sequenced and annotated, and each of them (with few exceptions, see below) contains *van* genes (Figure 1).

Thus far, the majority of GPA BGCs were found in members of the genus *Amycolatopsis* (Adamek et al., 2018), which belongs to the *Pseudonocardiaceae* family. Besides the vancomycin producers, other *Amycolatopsis* spp. produce avoparcin, decaplanin, nogabecin, ristocetin, teicoplanin aglycone-like GPA, norvancomycin, balhimycin, and chloroeremomycin. In their corresponding BGCs, *van* genes were found just upstream the genes coding for the StrR-like pathway-specific regulators (orthologues of *bbr* from balhimycin BGC, Figure 1). We excluded from this comparison the chloroeremomycin BGC from *Amycolatopsis orientalis* PA-42867 (?) (van Wageningen et al., 1998), which apparently was not completely covered with sequencing. Thus, three patterns for the organization of *van* genes are recognizable (Figure 1) in *Pseudonocardiaceae* GPA producers. In the producers of avoparcin, decaplanin, nogabecin, ristocetin, and teicoplanin-like aglycone GPA, the GPA BGCs carry *vanHAX* orthologues, but not *vanY* or *vanRS* orthologues. In vancomycin and norvancomycin producers, *vanY* orthologues are clustered with *vanHAX* ones. Balhimycin producer – *Amycolatopsis balhimycina* – possesses a BGC with *vanRS* (*vlnR_Ab_S_Ab_*) and *vanY* (*vanY_Ab_*) orthologues, but *vanHAX* orthologues (*vanH_Ab_A_Ab_X_Ab_*) were actually found 2 kbp away from balhimycin BGC (Schäberle et al., 2011; Frasch et al., 2015). Indeed, no cluster-situated *van* genes were found sequencing the genome of *Kibdelosporangium aridum* – the producer of kibdelins (Shearer et al., 1986) – which also belongs to *Pseudonocardiaceae* family (Figure 1).

Other known GPA BGCs are from *Actinoplanes* spp. (family *Micromonosporaceae*) and *Nonomuraea* spp. (family *Streptosporangiaceae*). *Act. teichomyceticus* and *Actinoplanes* sp. ATCC 53533 produce teicoplanin (Bardone et al., 1978) and UK-68,597 (Skelton et al., 1990), respectively. Teicoplanin BGC (named *tei*) contains *vanHAX* and *vanRS* orthologues (*tei7-6-5* and *tei2-3*, respectively) organized in two separate operons, but none *vanY* orthologue (Figure 1; Li et al., 2004; Yushchuk et al., 2020b). In contrast, UK-68,597 BGC contains a *vanH*, not contiguous *vanR* and *vanS*, and a *vanY* orthologue (Figure 1; Yim et al., 2014). In the genus *Nonomuraea*, *N. gerenzanenis* ATCC 39727 and *Nonomuraea* sp. ATCC 55076 produce A40926 (Goldstein et al., 1987) and the type V glycopeptide kistamicin (Naruse et al., 1993), respectively. In A40926 BGC (named *dbv*) (Sosio et al., 2003), *vanR* and *vanS* homologues (*dbv6* and *dbv22*, respectively) are not contiguous and GPA resistance is due to the expression of *vanY* orthologue (*dbv7*, Figure 1; Marcone et al., 2010, 2014; Binda et al., 2012; ). No *vanHAXY* genes are present in kistamicin BGC, although kistamicin BGC contains homologues of *vanS* and *vanR* named *kisG* and *kisB* (Nazari et al., 2017).

GPAs are also produced by few *Streptomyces* species (Figure 1). Interestingly, functional *van* genes were also found in *S. coelicolor*, which is not a GPA producer (Hong et al., 2004). A47934 BGC from *Streptomyces toyocaensis* NRRL 15009 contains *vanH_St_A_St_X_St_* and *vanR_St_S_St_* operons, together with *staO* and *staP* orthologues to *S. coelicolor vanJ* and *vanK*, respectively (Pootoolal et al., 2002). Pekiskomycin BGC from *Streptomyces* sp. WAC1420 contains *vanY*, *vanJ* as well as *vanHAX* and *vanRS* homologues, but pekiskomycin BGC from *Streptomyces* sp. WAC4229 lacks *vanRS* homologues (Thaker et al., 2013). No homologues of *van* genes were found in complestatin (type V GPA) BGC from *S. lavendulae* SANK 60477 (?) (Chiu et al., 2001), although this antibiotic possesses a moderate antibacterial activity. However, complestatin was shown to inhibit the fatty acid biosynthesis in Gram-positive bacteria (Kwon et al., 2015), therefore the producer may require no cell wall remodeling for complestatin self-resistance. Finally, feglymycin BGC from *Streptomyces* sp. DSM11171 (Figure 1) encodes for a 13-mer peptide antibiotic acting on bacterial cell wall biosynthesis by inhibiting MurA and MurC. Albeit the structure and the mode of action of feglymycin differs from the ones of GPAs, feglymycin BGC shares a high level of similarity with GPA BGCs (Gonsior et al., 2015; Yushchuk et al., 2020a), including the presence of *vanRS*-like genes – *fegM* and *fegN*.

To conclude, CA915, CA37, and CA878 GPA BGCs (Banik et al., 2010), which were sequenced from metagenomics samples, contain *vanHAX*, *vanY* and *vanRS* homologues, whereas none *van* gene was found in other metagenome-derived GPA BGCs as TEG and VEG (Banik and Brady, 2008; Figure 1).

## Updating On What Is Known About The *In Vivo* Function Of *Van* Genes In Gpa-Producing Strains

**Figure 2:**
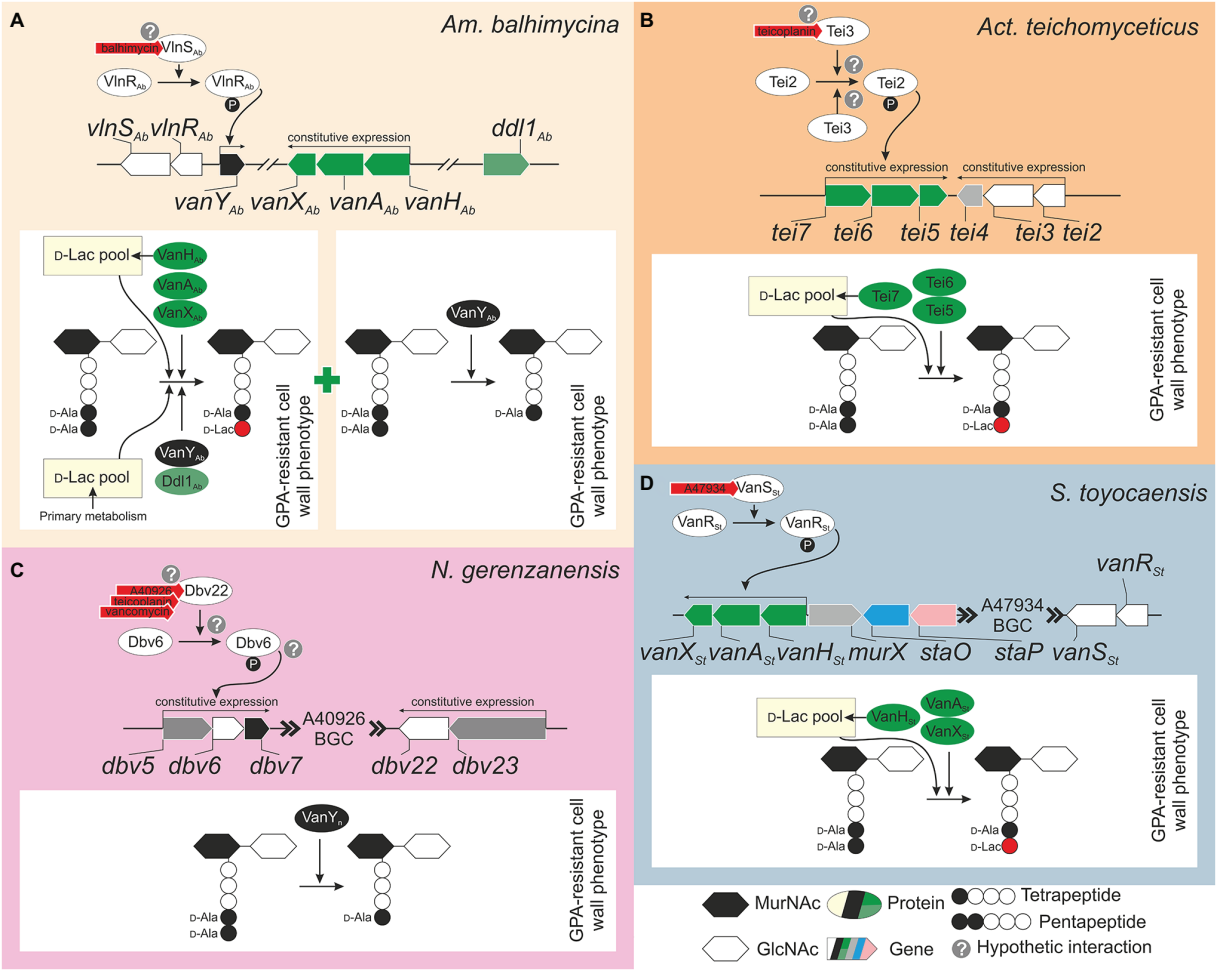
Knowledge-based conceptual schemes for the GPA self-resistance mechanisms and their regulation in model GPA-producers: **(A)** *Am. balhimycina*, the producer of balhimycin, **(B)** *Act. teichomyceticus*, the producer of teicoplanin, **(C)** *N. gerenzanensis*, the producer of A40926, and **(D)** *S. toyocaensis*, the producer of A47934. Please refer to main text for more details on genes involved in GPA self-resistance. Please note that *tei4* in *Act. teichomyceticus*, *dbv5* and *dbv23* in *N. gerenzanensis*, and *murX* in *S. toyocaensis*, colored in light gray, are not *van* gene orthologues, but they are reported since they are close to *van* genes or part of their operons.

Although *van* genes were found in multiple GPA BGCs, only for few of them the function was experimentally proven. Balhimycin resistance in *Am. balhimycina* is likely the most deeply investigated model among GPA producers (Figure 2A). *vanH_Ab_A_Ab_X_Ab_*, that is located outside the BGC (Figure 1), was shown to be constitutively expressed through all the periods of growth and during balhimycin production (Schäberle et al., 2011). Deletion of *vanH_Ab_A_Ab_X_Ab_* genes makes *Am. balhimycina* significantly more sensitive to its own product, decreasing its MIC from 5 to 0.25 mg/ml, and causing an earlier expression of the BGC-situated *vanY_Ab_* (Frasch et al., 2015). However, *vanY_Ab_* itself does not play a decisive role in GPA-resistance since its deletion did not alter the GPA resistance phenotype (Frasch et al., 2015). Double *vanH_Ab_A_Ab_X_Ab_* and *vanY_Ab_* knocked-out mutants showed the same GPA resistance phenotype as Δ*vanH_Ab_A_Ab_X_Ab_*. PG precursors ending in d-Ala-d-Lac were still found in the single Δ*vanH_Ab_A_Ab_X_Ab_* and in the double Δ*vanH_Ab_A_Ab_X_Ab_* Δ*vanY_Ab_* mutants together with d-Ala-d-Ala ending PG precursors and tetrapeptides (Frasch et al., 2015). The residual GPA resistance in these mutants is probably due to an accessory Ddl1_Ab_, a putative d-Ala-d-Lac ligase encoded in the genome of *Am. balhimycina*, which shares 72% of amino acid sequence identity with VanA_Ab_ (Frasch et al., 2015). Ddl1_Ab_ might add d-Lac to the tetrapeptide PG precursors generated by the d,d-carboxypetidase VanY_Ab_ (although the presence of some other d-Ala-d-Ala carboxypeptidases encoded in the genome cannot be completely ruled out considering the resistant phenotype of the Δ*vanY_Ab_* mutant). In the absence of VanH_Ab_, d-Lac for this reaction is probably obtained from the primary metabolic pool. Expression of *vanH_Ab_A_Ab_X_Ab_* was demonstrated to be independent from the BGC-situated regulator *vlnR_Ab_* (Kilian et al., 2016). However, VlnR_Ab_ is important for the activation of the BGC-situated *vanY_Ab_* expression (Kilian et al., 2016). Heterologous expression of *vlnR_Ab_S_Ab_* in *S. coelicolor* Δ*vanRS* mutants indicated that both VlnR_Ab_ and VlnS_Ab_ are active and able to replace their counterparts VanR and VanS, which in *S. coelicolor* control the expression of *vanHAX* genes in response to vancomycin (Hong et al., 2004), restoring resistance to both balhimycin and teicoplanin in the complemented strains (Kilian et al., 2016). Overall, it seems that the BGC-situated *vlnR_Ab_S_Ab_-vanY_Ab_* regulatory circuit is functional, but does not play a major role in balhimycin resistance, which is mostly determined by *vanH_Ab_A_Ab_X_Ab_* expression. It would be interesting to test GPA resistance in *ddl1_Ab_* knocked-out mutant generated in *Am. balhimycina* Δ*vanH_Ab_A_Ab_X_Ab_* Δ*vanY_Ab_* to better understand the role of this accessory ligase and its connection with the d,d-carboxypetidase activity of VanY_Ab_ (or of some other still-unknown carboxypeptidases).

Differently from *Am. balhimycina*, in *Act. teichomyceticus vanHAX* orthologues – *tei7-6-5* – are located within the *tei* BGC together with *vanRS* orthologues – *tei2-3* (Figure 2B). *tei7-6-5* expression determines the production of PG precursors ending in d-Ala-d-Lac, conferring a GPA-resistant phenotype to *Act. teichomyceticus* (Beltrametti et al., 2007; Binda et al., 2018). Interestingly, the expression of *tei7-6-5* operon is constant during the growth curve and in teicoplanin production conditions (Beltrametti et al., 2007; Yushchuk et al., 2019) and the VanX d,d-dipeptidase activity was detectable in cellular extracts independently from the addition of teicoplanin (Binda et al., 2018). One probable reason for the constitutive expression of *tei7-6-5* is the non-inducibility of the sensor histidine kinase Tei3, due to its point mutations previously known to confer a constitutive kinase activity to *S. coelicolor* VanS (Beltrametti et al., 2007). Also, the expression of *vanRS* orthologues – *tei2-3* – was also found constitutive under teicoplanin production conditions (Yushchuk et al., 2019) and these genes are co-expressed with *tei4* – coding for a dehydrofolate reductase with no obvious role in teicoplanin-resistance (Yushchuk et al., 2020b). Moreover, *tei2-3-4* expression is independent from *tei* cluster-encoded transcriptional regulators – Tei15* and Tei16* (Yushchuk et al., 2019, 2020b). Constitutive expression of *tei2-3-4* could be granted by *tei2* promoter, which was shown to be highly active in *Act. teichomyceticus*, starting from the very early stage of spore germination (Yushchuk et al., 2020b). More investigations are required for a complete understanding of teicoplanin-resistance in *Act. teichomyceticus*. Study of the Tei3 properties is among the most interesting tasks.

In *N. gerenzanensis* producing the teicoplanin-like A40926, *vanHAX* orthologues were not found neither in the BGC nor in the genome (D’Argenio et al., 2016). The only known mechanism of resistance relies on the action of VanY_n_, whose coding gene (*dbv7*) is within the *dbv* BGC (Figure 2C) and whose knockout abolishes the resistance phenotype (Marcone et al., 2010, 2014; Figure 2C). VanY_n_ is a d,d-carboxypeptidase that cleaves the last d-Ala from pentapeptide PG precursors generating tetrapeptides, drastically reducing GPA affinity for cellular targets (Binda et al., 2012). A l,d-transpeptidase (Ldt) then uses the tetrapeptide acyl donors supplied by VanY_n_ to synthetize the mature cell wall (Hugonnet et al., 2014). The role of this protein and its features that assimilate/distinguish it from enterococcal VanY and from VanY_Ab_ were investigated in detail (Marcone et al., 2010, 2014; Binda et al., 2012, 2013). Less clear is the regulatory circuit governing *dbv7* expression. Direct VanY_n_ carboxypeptidase activity measurement in *N. gerenzanensis* growing with the addition of different GPAs, unambiguously showed that VanY_n_ activity is induced by vancomycin, teicoplanin, and A40926 (Binda et al., 2018). *vanRS* homologues – *dbv6* and *dbv22* – are present in the *dbv* BGC, but the knockout of *dbv6* did not exert any influence on A40926 production and growth of *N. gerenzanensis* (Lo Grasso et al., 2015). Unfortunately, the GPA resistance phenotype of this mutant was not described. On the other side, transcriptional analysis of *dbv* genes indicated that the expression of *dbv5-6-7* and *dbv23-22* operons is rather constitutive (Alduina et al., 2007). Although the presence of other GPA-sensitive TCS beyond the borders of A40926 BGC cannot be ruled out, role of Dbv6 and Dbv22 in *N. gerenzanensis* A40926 self-resistance merits further investigations.

Finally, *S. toyocaensis* possesses, perhaps, the most straightforward resistance mechanism among all the investigated GPA producers (Figure 2D), reminding the situation in *S. coelicolor* (Hong et al., 2004). The BGC-located *vanH_St_A_St_X_St_* operon was shown to be crucial for A47934 resistance and *vanA_St_* knockout made *S. toyocaensis* completely sensitive to A47934 (Pootoolal et al., 2002). At the same time, *vanHAX*-genes from the vancomycin producer *Am. orientalis* C329.2 were able to restore A47934 resistance phenotype in the knocked-out mutant (Pootoolal et al., 2002). Functions of VanR_St_ and VanS_St_ (both present in the A47934 BGC, Figures 1, 2D) were also studied in detail, showing that VanS_St_ has a remarkable specificity for A47934 and it is unable to sense teicoplanin or vancomycin (Koteva et al., 2010; Novotna et al., 2016). Moreover, also the interaction between VanR_St_ and VanS_St_ was found to be very specific, since VanR_St_ could not be phosphorylated by a non-cognate sensor-histidine kinase (Novotna et al., 2016). The roles (if there are any) of *staP* and *staO* (orthologues of *S. coelicolor vanK* and *vanJ*) in *S. toyocaensis* A47934 self-resistance were not investigated, thus the importance of these auxiliary resistance genes remains to be proved.

## Outlook

Soil GPA producers are considered the putative source of GPA resistance determinants, which might had been recruited and differently combined in pathogens. The goal of this mini review is to update the knowledge on the occurrence and role of *van* genes in producing microorganisms. It emerges that more *in silico*, *in vitro*, and *in vivo* investigations on their function and regulation are required to shed light on the intriguing issue of their origin and role. Overall, a detailed phylogenetic analysis would be useful to illuminate the evolution of GPA-resistant determinants in GPA producers and from them to pathogens. A recent pioneering work on the reconstruction of GPA BGC phylogeny (Waglechner et al., 2019) reported on the possible origin and evolution of GPA cluster-situated *van*-genes. According to these authors, *vanA* had likely originated within *Amycolatopsis* genus, whereas *vanH*, *vanX*, and *vanRS* within *Actinoplanes*; and *vanY* probably originated within genus *Nonomuraea* and it was then distributed among GPA BGCs by multiple transfer events. Combination of these genes in pathogens is today determining the urgent clinical need for new drugs to combat multi-drug resistant Gram-positive pathogens.

## Author Contributions

EB and OY collected data and papers and co-wrote the review. OY prepared the figures. FM and EB supervised the work.

## Conflict of Interest

The authors declare that the research was conducted in the absence of any commercial or financial relationships that could be construed as a potential conflict of interest.
